# Nuclear factor erythroid 2 related factor 2, tumor necrosis factor alpha, interleukin-40, and endorepellin levels in patients undergoing coronary artery bypass grafting

**DOI:** 10.1590/1806-9282.20251054

**Published:** 2026-04-20

**Authors:** Reşat Dikme, İsmail Koyuncu, Mehmet Salih Aydın

**Affiliations:** 1 Harran University, Vocational School of Health Services, Dialysis Program – Şanlıurfa, Turkey.; 2 Harran University, Faculty of Medicine, Medicinal Biochemistry – Şanlıurfa, Turkey.; 3 Harran University, Faculty of Medicine, Department of Cardiovascular Surgery – Şanlıurfa, Turkey.

**Keywords:** Coronary artery disease, Oxidative Stress, Tumor necrosis factor-alpha, Interleukins, Endorepellin

## Abstract

**OBJECTIVE:**

The aim of this study was to evaluate the levels of nuclear factor erythroid 2 related factor 2, tumor necrosis factor alpha, interleukin-40, and endorepellin in patients undergoing coronary artery bypass grafting and explore their potential diagnostic roles.

**METHODS:**

The study involved 43 coronary artery disease patients undergoing coronary artery bypass grafting and 43 age- and sex-matched healthy controls. Plasma levels of nuclear factor erythroid 2 related factor 2, tumor necrosis factor alpha, interleukin-40, and endorepellin were quantified by enzyme-linked immunosorbent assay. Statistical analyses were conducted using SPSS v25.0, with significance set at p<0.05.

**RESULTS:**

Nuclear factor erythroid 2 related factor 2 levels were significantly reduced, while tumor necrosis factor alpha and interleukin-40 were elevated in coronary artery disease patients (p<0.001, p=0.003, p=0.001). Endorepellin levels did not differ significantly (p=0.120). Nuclear factor erythroid 2 related factor 2 correlated inversely with tumor necrosis factor alpha and interleukin-40; tumor necrosis factor alpha and interleukin-40 showed a positive correlation. Regression identified nuclear factor erythroid 2 related factor 2 as a tumor necrosis factor alpha predictor. Principal component analysis distinguished patients from controls based on biomarker profiles. These findings reflect enhanced inflammation and impaired antioxidant defense in coronary artery disease. Interleukin-40 may serve as a novel inflammatory biomarker; endorepellin's unchanged levels suggest localized vascular effects.

**CONCLUSION:**

Nuclear factor erythroid 2 related factor 2, tumor necrosis factor alpha, and interleukin-40 emerged as significant biomarkers reflecting the inflammatory and oxidative stress mechanisms associated with coronary artery disease. Interleukin-40 and endorepellin may offer new insights into disease mechanisms and warrant further research.

## INTRODUCTION

Recent studies emphasize the role of nuclear factor erythroid 2 related factor 2 (NRF-2), a key antioxidant regulator, and tumor necrosis factor alpha (TNF-α), a major proinflammatory cytokine, in ­coronary artery disease (CAD) pathophysiology^1,2^. NRF-2 downregulation increases oxidative stress and vascular dysfunction^3,4^, while TNF-α is strongly associated with CAD progression and elevated risk^5^.

Although NRF-2 and TNF-α have been widely studied, the cardiovascular significance of newly identified molecules such as interleukin-40 (IL-40) and the extracellular matrix fragment endorepellin remains insufficiently explored^6,7^. IL-40 is a recently discovered pro-inflammatory cytokine, primarily linked to B cells and humoral immune responses^8^. It is upregulated in various chronic inflammatory diseases, correlates with ­disease activity, and contributes to acute inflammation, suggesting its potential as a biomarker of systemic inflammatory states^8^.

Endorepellin, the LG3 domain of perlecan, exerts anti-angiogenic effects via autophagy and apoptosis in endothelial cells^7,9^. It is released into circulation during inflammation or tissue injury. Elevated levels have been reported in chronic allograft nephropathy and acute cardiac rejection, indicating its potential as a marker of inflammation and tissue remodeling^7,9^. However, its role in CAD and circulating levels in coronary artery bypass grafting (CABG) patients remains poorly defined.

This study aims to assess preoperative plasma levels of NRF-2, TNF-α, IL-40, and Endorepellin in patients ­undergoing CABG for CAD, compared to healthy controls, to explore their diagnostic potential and roles in CAD pathophysiology.

## METHODS

### Study population and blood sampling

This study included 43 patients (Group 1) scheduled for elective CABG due to severe coronary artery disease and 43 age- and sex-matched healthy volunteers (Group 2). Patients with acute myocardial infarction, unstable angina, active infection, malignancy, rheumatologic disease, or end-stage renal or hepatic failure were excluded. All patients were in stable clinical condition. Venous blood samples were obtained preoperatively from patients and at enrollment from controls, collected into ethylenediaminetetraacetic acid (EDTA) tubes, centrifuged at 5,000 rpm for 10 min, and stored at −80°C until analysis.

### Laboratory analyses

Plasma concentrations of NRF-2 (FNTEST Human NFE2L2–ELISA Kit, range: 0.156–10 ng/mL), TNF-α (FNTEST Human TNF-α–ELISA Kit, EH0302, range: 15.625–1,000 pg/mL), IL-40 (FNTEST Human IL-40 ELISA Kit, EH5091, range: 31.25–2,000 pg/mL), and endorepellin (RayBiotech Human Endorepellin ELISA Kit, ELH-ENRPN, range: 0.08–25 ng/mL) were quantitatively measured using the enzyme-linked immunosorbent assay (ELISA) method in accordance with the manufacturers’ instructions.

### Statistical analysis

Statistical analyses were conducted using IBM SPSS Statistics version 25.0 (IBM Corp., Armonk, NY, USA). Data distribution was evaluated with the Shapiro-Wilk test. Non-normally distributed continuous variables were compared using the Mann-Whitney U test. Spearman's rho was used for correlation analysis, and multiple linear regression assessed the strength and direction of associations. Principal component analysis (PCA) was performed to evaluate data structure and visualize group separation. Receiver operating characteristic (ROC) analysis determined the diagnostic performance of each biomarker. A p<0.05 was considered statistically significant.

## RESULTS

The demographic and clinical characteristics of the patient and control groups, along with surgical details, are summarized in Table 1. Regarding comorbidities, 16 patients (37.20%) had diabetes mellitus, 7 (16.27%) had hypertension, and 8 (18.60%) had both conditions. Twelve patients (27.90%) had no accompanying comorbidities. Additionally, 17 patients (39.53%) were active smokers. Regarding surgical procedures, 10 patients (23.25%) underwent CABG with two grafts (CABGX2), 18 patients (41.86%) received three grafts (CABGX3), and 15 patients (34.88%) received four grafts (CABGX4).

**Table 1:** Characteristics of patients and control groups.

Variable		Patient group	Control group	p
Age (years)		62.43±6.51	59.27±9.74	>0.05
Height (cm)		164.45±8.67	166.78±9.13	>0.05
Weight (kg)		78.92±11.46	80.85±13.34	>0.05
BSA (m²)		1.83±0.24	1.85±0.19	>0.05
Sex				
	Female, n	18 (41.86%)	16 (37.20%)	>0.05
	Male, n	25 (58.13%)	27 (62.80%)	>0.05
Comorbidities				
	Diabetes mellitus	16 (37.20%)	0	
	Hypertension	7 (16.27%)	0	
	Diabetes mellitus+hypertension	8 (18.60%)	0	
	No comorbidities	12 (27.90%)		
Smoking				
	Smoking	17 (39.53%)	0	
	No smoking	26 (60.47%)	0	
CABG				
	CABGX2	10 (23.25%)	0	
	CABGX3	18 (41.86%)	0	
	CABGX4	15 (34.88%)	0	

CABG: coronary artery bypass grafting; BSA: body surface area.

As the variables did not exhibit a normal distribution in either group, a non-parametric test (Mann-Whitney U) was applied. Plasma biomarker levels in the patient and control groups are summarized in Table 2. Accordingly, TNF-α and IL-40 ­levels were significantly higher in the patient group compared to the control group (p=0.003 and p=0.001, respectively), while NRF-2 levels were significantly lower (p<0.001). Endorepellin levels did not differ significantly between the groups (p>0.05).

**Table 2:** Plasma biomarker levels in patient and control groups.

Variable	Group	Mean±SD	Median (IQR)	P	r	Z score
NRF-2 (ng/mL)	Patient	2.03±0.39	2.11 (1.99–2.13)	<0.001*	0.60	-4.12
	Control	2.72±0.33	2.75 (2.67–2.84)			
TNF-α (pg/mL)	Patient	269.14±120.12	249.75 (189–351)	0.003*	0.45	-2.96
	Control	163.91±89.87	156.75 (102.6–199.8)			
IL-40 (pg/mL)	Patient	228.59±136.47	190.48 (170–281.6)	0.001*	0.52	-3.41
	Control	115.82±38.12	111.46 (105.6–114.7)			
Endorepellin (ng/mL)	Patient	2.05±0.62	1.92 (1.68–2.06)	0.120	0.20	-1.56
	Control	1.85±0.83	1.62 (1.35–2.04)			

p<0.05 was considered statistically significant. The Mann-Whitney U test was applied for comparisons of non-parametric variables between groups. r: Indicates effect size, calculated as the rank-biserial correlation coefficient. r=|Z|/√N (n: total sample size). Effect sizes were interpreted as follows: 0.1=small, 0.3=medium, and ≥0.5=large. Z-scores represent the standardized differences between groups obtained from non-parametric tests. Effect sizes (r), calculated as rank-biserial correlation coefficients, provide an estimate of the clinical magnitude of differences independent of sample size. Medium effect sizes for TNF-α and IL-40 indicate that these biomarkers are not only statistically significant but also clinically meaningful in reflecting systemic inflammation in CAD. In contrast, the small effect size for endorepellin suggests limited systemic impact, supporting the notion of a predominantly localized vascular role. TNF-α: tumor necrosis factor alpha; IL-40: interleukin-40.

* Indicates statistical significance (p<0.05). SD: standard deviation; IQR: interquartile range.

In the patient group, NRF-2 levels (2.03±0.39 ng/mL, median: 2.11) were lower compared to the control group, and this difference was found to be statistically significant (p<0.001). TNF-α (269.14±120.12 pg/mL, median: 249.75) and IL-40 (228.59±136.47 pg/mL, median:190.48) levels were higher in the patient group compared to controls, and these differences were also statistically significant (TNF-α: p=0.003; IL-40: p=0.001). In contrast, endorepellin levels (patient group: 2.05±0.62 ng/mL; control group: 1.85±0.83 ng/mL) did not show a statistically significant difference between groups (p=0.120). No significant differences were observed in biomarker levels based on comorbidities (diabetes, hypertension), smoking status, or the number of grafts used in CABG (p>0.05). The evaluation of effect sizes confirmed that the differences observed for TNF-α and IL-40 were not only statistically significant but also clinically relevant, whereas the small effect size for endorepellin indicated limited systemic significance.

Correlation analysis revealed a significant negative correlation between NRF-2 and TNF-α (r=-0.412, p=0.0005) and between NRF-2 and IL-40 (r=-0.282, p=0.0197), while TNF-α and IL-40 were positively correlated (r=0.45, p<0.001). No other correlations reached statistical significance.

Regression models (Table 3) showed that NRF-2 and TNF-α significantly predicted each other in a bidirectional inverse manner (p=0.007), supporting the possibility of their opposing roles in disease pathophysiology. A borderline association was noted between IL-40 and NRF-2 (p=0.054). Endorepellin did not show a significant effect in any model (p>0.05). The negative β coefficients indicate that a decrease in NRF-2 levels is associated with an increase in TNF-α, reflecting the biological interplay between impaired antioxidant defense and heightened inflammatory activity in CAD.

**Table 3:** Results of regression models for each variable.

Dependent variable	Independent variable	β (coefficient)	P
NRF-2	TNF-α	-0.0013	0.007
NRF-2	IL-40	-0.0010	0.054
NRF-2	Endorepellin	0.0228	0.678
TNF-α	NRF-2	-81.05	0.007
IL-40	NRF-2	-54.80	0.054
Endorepellin	All variables	–	>0.5

NRF-2: nuclear factor erythroid 2 related factor 2; TNF-α: tumor necrosis factor alpha; IL-40: interleukin-40.

PCA, applied to reduce multidimensional data and identify group-specific patterns, demonstrated that the first two principal components (PC1: 38.4%, PC2: 25.6%) explained approximately 64% of the total variance, indicating a clear separation between the patient and control groups based on biomarker profiles.

In the ROC analysis performed to assess the discriminative power of the biomarkers, the following area under the curve (AUC) values were obtained: IL-40: 0.80, TNF-α: 0.75, Endorepellin: 0.54, and NRF-2: 0.00. It should be noted that the AUC value of 0.00 for NRF-2 does not indicate a lack of discriminative power but rather reflects an inverse relationship, where lower levels of NRF-2 are associated with CAD. In practical terms, this corresponds to an effective discriminative ability equivalent to an AUC of 1.00, supporting NRF-2 as a negative biomarker.

## DISCUSSION

### Nuclear factor erythroid 2 related factor 2 and cardioprotective mechanisms

NRF-2 levels were significantly reduced in CAD patients, reflecting impaired antioxidant defense. As a key transcription factor, NRF-2 modulates oxidative stress and inflammation, and its downregulation contributes to disease progression^1,10^ Experimental models support its cardioprotective role through endothelial preservation, reduced apoptosis, and cytokine suppression^11^.

Our findings highlight the elevated oxidative burden and diminished NRF-2 response in CAD. Enhancing NRF-2 activity via pharmacological agents or antioxidants has shown potential to mitigate cardiovascular oxidative damage^12,13^. Preoperative NRF-2 measurement may help identify patients at high risk and guide strategies to reduce perioperative oxidative injury.

### Tumor necrosis factor alpha and atherosclerotic inflammation

TNF-α is a central mediator of inflammation in atherosclerosis and CAD^14^. Its elevation contributes to endothelial dysfunction, plaque formation, and destabilization^15^. Higher TNF-α levels have been reported in CAD patients, especially during acute coronary events^16^. The elevated TNF-α levels observed in our CAD cohort confirm its role as a marker of persistent subclinical inflammation, in line with previous studies. These results underscore the potential benefit of anti-inflammatory strategies in CAD management. The regression results provide further support for this mechanistic link. Specifically, the inverse predictive relationship between NRF-2 and TNF-α suggests that downregulation of NRF-2 not only reduces antioxidant capacity but may also drive the upregulation of pro-inflammatory mediators. This highlights the dual clinical relevance of NRF-2, both as a marker of oxidative imbalance and as a potential upstream regulator of inflammatory pathways. The borderline association observed between NRF-2 and IL-40 further suggests that B-cell-related inflammatory responses may also be modulated by redox status, warranting investigation in larger cohorts.

### Potential role of interleukin-40 in the cardiovascular system

Various interleukins contribute to CAD pathogenesis and may hold diagnostic and prognostic value^17,18^. IL-40, a recently identified B cell-associated cytokine, is linked to autoimmune and inflammatory diseases^6,19,20^. Although its role in CAD remains unclear, IL-40 has diagnostic potential, as shown by its elevation in rheumatoid arthritis^21,22^. Its reduction following anti-TNF therapy suggests regulation by TNF-α^22^.

In our study, IL-40 levels were significantly higher in CAD patients, correlating positively with TNF-α. These findings support a potential role for IL-40 in vascular inflammation, possibly through B cell activation and TNF-α-mediated signaling^23^. As this is the first clinical study assessing IL-40 in CAD, our results highlight its promise as a novel inflammatory biomarker, independent of traditional risk factors, warranting validation in future research.

### Endorepellin and angiogenesis regulation

Endorepellin exerts anti-angiogenic effects by inhibiting Hyaluronan Synthase 2 and inducing autophagy in endothelial cells, potentially modulating vascular remodeling and cellular stress responses^7,9^. Although its role in cardiovascular disease remains unclear, it may influence plaque neovascularization or impair collateral vessel formation, suggesting dual pathophysiological roles^24^. In our study, endorepellin levels did not significantly differ between CAD patients and controls, limiting its utility as a systemic biomarker. Its local vascular effects may still impact graft outcomes, warranting tissue-level research. Taken together, the analysis of effect sizes suggests that TNF-α and IL-40 reflect clinically relevant inflammatory processes in CAD, whereas endorepellin may exert its influence primarily at the local vascular level rather than as a systemic biomarker. Although systemic differences were not detected, endorepellin may still modulate localized vascular processes such as endothelial autophagy, plaque neovascularization, and graft remodeling. These site-specific effects highlight the need for future tissue-level or mechanistic studies to fully elucidate its role in CAD.

### Receiver operating characteristic analysis

ROC analysis revealed strong diagnostic potential for IL-40 (AUC=0.80) and TNF-α (AUC=0.75). Although NRF-2 initially yielded an AUC of 0.00, this reflects its inverse correlation with CAD; thus, its effective discriminative power can be interpreted as 1.00. This underscores NRF-2's relevance as a negative biomarker associated with impaired antioxidant defense. In contrast, endorepellin demonstrated limited diagnostic utility (AUC=0.54), suggesting a more localized vascular role rather than systemic involvement. This finding indicates that NRF-2 functions as a negative biomarker, with reduced plasma levels serving as an indicator of CAD. The apparent AUC of 0.00 in ROC analysis represents an inverse classification, which, when corrected, highlights NRF-2's strong discriminative capacity. Therefore, although its interpretation requires caution, NRF-2 may provide valuable diagnostic information as a reverse biomarker of impaired antioxidant defense.

## CONCLUSION

This study demonstrates that CAD involves both enhanced inflammation and impaired antioxidant defense. Plasma levels of TNF-α and IL-40 were significantly elevated in CAD patients, while NRF-2 was markedly reduced, indicating disrupted redox homeostasis. Although endorepellin levels were not significantly elevated, its localized vascular effects may still be clinically relevant. ROC analysis confirmed the strong diagnostic potential of IL-40, TNF-α, and especially NRF-2, despite its inverse association. These biomarkers were unaffected by comorbidities, suggesting disease-specific alterations. Collectively, NRF-2 and IL-40 may serve as novel biomarkers and therapeutic targets, supporting their use in CAD risk stratification and intervention strategies.

## Data Availability

The datasets generated and/or analyzed during the current study are available from the corresponding author upon reasonable request.
